# Measuring Gratitude in Germany: Validation Study of the German Version of the Gratitude Questionnaire-Six Item Form (GQ-6-G) and the Multi-Component Gratitude Measure (MCGM-G)

**DOI:** 10.3389/fpsyg.2020.590108

**Published:** 2020-10-08

**Authors:** Matthias F. C. Hudecek, Nicole Blabst, Blaire Morgan, Eva Lermer

**Affiliations:** ^1^University of Regensburg, Regensburg, Germany; ^2^FOM University of Applied Sciences for Economics and Management, Munich, Germany; ^3^Department of Psychology, University of Worcester, Worcester, United Kingdom; ^4^Center for Leadership and People Management, LMU Munich, Munich, Germany

**Keywords:** gratitude, Gratitude Questionnaire-Six Item Form, Multi-Component Gratitude Measure, validation study, confirmatory factor analysis, cultural measurement invariance

## Abstract

The Gratitude Questionnaire-Six Item Form (GQ-6; McCullough et al., 2002) is a well-established instrument for measuring gratitude. Recently, the Multi-Component Gratitude Measure (MCGM) was developed as a more holistic approach (Morgan et al., 2017). While the GQ-6 mainly focuses on the emotional component of gratitude, the MCGM encompasses conceptual, attitudinal, and behavioral aspects. As of today, there is no validated German measure for gratitude. In order to close that research gap, the present study focused on validating the German versions of the GQ-6 (GQ-6-G) and of the MCGM (MCGM-G). In addition, multi-group comparisons were conducted to test for cultural measurement invariance. Construct validity was tested similar to original validation studies of the two scales focusing on affect, well-being, empathy, anxiety and depression. The online survey was completed in random order by 508 participants. The one-factor model of the GQ-6-G and the hierarchical structure of the MCGM-G could be replicated. However, the model fit of the Gratitude Questionnaire was significantly better after eliminating one item (GQ-5-G). Multi-group comparisons revealed cultural measurement invariance was established for the GQ-5-G and partial measurement invariance for five of six factors of the MCGM-G, respectively. Reliability analyses revealed good internal consistency for both instruments, and measures for criterion-related and discriminant validity have shown hypothesized relationships. Thus, the GQ-5-G and the MCGM-G are two instruments with good reliability and validity for measuring gratitude in Germany.

## Introduction

Gratitude is a moral virtue that has universal and timeless presence in human life and has been discussed upon an intellectual level for centuries by philosophers and scholars (McCullough et al., 2002). In everyday lived experiences of gratitude, one can easily bring to mind an occasion that has warranted the simple expression of “thank you.” Indeed, knee-jerk expressions of gratitude have arguably become an unconscious part of interacting with others: you thank the person holding the door open for you, you say “thank you” to your colleague for sending an important email, and you express your gratitude after sneezing and receiving a “bless you” from your partner. However, a now diverse field of psychological literature on gratitude has shown it to be much more complex than simple expressions of “thank you.” The complexity of this construct is mirrored in the fact that there is no one agreed definition of the construct. Thus, gratitude can be understood and experienced in multiple ways (Morgan et al., 2017). According to Wood et al. (2010), gratitude can be seen as a trait-like disposition as well as a temporary state, such as a positive mood. Gratitude as an emotion is directed toward appreciating valuable help or actions from others (McCullough et al., 2001). However, there is also some evidence that gratitude can be associated with negative features, e.g., feelings of obligation, guilt, or embarrassment (Morgan et al., 2014). At the dispositional level, gratitude can be understood as “part of a wider life orientation toward noticing and appreciating the positive in the world” (Wood et al., 2010, p. 891) and has been shown to be distinct from facets of personality and other positive psychology constructs such as optimism and hope (Wood et al., 2008). This suggests that a latent grateful disposition exists.

At present there exist four well-established different measures to assess gratitude (Morgan et al., 2017; Lermer, 2019). The firstly developed measure of gratitude, the unifactorial Gratitude Questionnaire-Six Item Form (GQ-6), mainly focuses on the emotional component of gratitude (McCullough et al., 2002). In that context, gratitude was defined as “a generalized tendency to recognize and respond with grateful emotion to the roles of other people’s benevolence in the positive experiences and outcomes that one obtains” (McCullough et al., 2002, p. 112). The GQ-6 consists of six items (e.g., “I have so much in life to be thankful for”) that measure span, frequency, intensity, and density of gratitude (McCullough et al., 2002). In contrast, the Gratitude, Resentment and Appreciation Test (GRAT; Watkins et al., 2003) provides a broader view of gratitude. It consists of three subscales: sense of abundance (e.g., “There never seems to be enough to go around and I’m always coming up short”), simple appreciation (e.g., “I think that it’s important to ‘Stop and smell the roses”’), and appreciation of others (e.g., “I feel deeply appreciative for the things others have done for me in my life”). The third measure, the Appreciation scale, developed by Adler and Fagley (2005), conceptualizes and assesses gratitude as a subordinate facet of the broader construct of appreciation. Originally, appreciation was defined to measure something distinct from gratitude (Adler and Fagley, 2005), but Wood et al. (2008) have reported that gratitude and appreciation are a single-factor trait rather than distinct constructs. This broader appreciation scale comprises eight subscales: a focus on what one has (“have focus”), awe, ritual, present moment, self/social comparison, gratitude, loss/adversity, and interpersonal. After more than 10 years of not having any significant developments measuring gratitude, Morgan et al. (2017) introduced the Multi-Component Gratitude Measure (MCGM). The MCGM offers a holistic approach to gratitude measurement, as the authors consolidated the existing definitions. In total, the MCGM consists of four components. In addition to the original *emotional* aspect of gratitude, already proposed by McCullough et al. (2002), they added an *attitudinal* and *behavioral* aspect in their understanding of gratitude. The attitude component focuses on when to show gratitude and how gratitude is perceived in the context of values (e.g., “I believe it is important to thank people sincerely for the help they give me”). The behavioral component consists of the expression as well as lack of expression of gratitude toward one’s benefactors and of a more spiritual connection toward being thankful (e.g., “I stop to recognize all the good things I have in my life”). In addition, Morgan et al. (2017) proposed that people can understand and experience gratitude in various ways. Thus, the *conceptual* aspect was added to capture cognitions about gratitude. A fifth measure of gratitude was developed by Bernabé-Valero et al. (2014). The Gratitude Questionnaire-20 Items (G-20) captures four dimensions of gratitude (interpersonal gratitude, gratitude before suffering, the recognition of personal gifts in personal experience, and the expression of gratitude) using 20 items and was established using a sample of 330 Spanish undergraduates. As of today, the instrument is only available in Spanish and was recently validated with an Argentinian sample that supports the four-factor structure (Klos et al., 2020).

Early research found that being grateful is associated with a happier and more optimistic outlook on life (Watkins et al., 2003) and the tendency to overcome adversity more easily (Emmons and McCullough, 2003). Consequently, gratitude was shown to be related with higher subjective well-being (Emmons and McCullough, 2003) and life satisfaction (Peterson et al., 2007). Another well-established finding is the association of gratitude with lower risks of depression and reduced anxiety (Kendler et al., 2003; Froh et al., 2007, 2011). Further health benefits were reported concerning reduced stress (Wood et al., 2008) and improved sleep patterns (Emmons and McCullough, 2003; Wood et al., 2009). In addition, gratitude relates to interpersonal benefits, such as the ability to form and maintain stronger interpersonal bonds (Algoe, 2012; Bartlett et al., 2012) and promoting prosocial behaviors (Bartlett and DeSteno, 2006).

In order to conduct comparable and replicable studies on gratitude in Germany, it is vital to provide reliable and valid measuring tools. As of today, there is no validated German instrument measuring gratitude. Thus, the present study aimed to validate the most frequently used unidimensional measure, the GQ-6 (McCullough et al., 2002), and the newest holistic measure of gratitude, the Multi-Component Gratitude Measure (MCGM; Morgan et al., 2017). The GQ-6 has already been validated in several other languages including Hungarian (Tamás et al., 2014), Dutch (Jans-Beken et al., 2015), Chinese (Chen et al., 2008), Portuguese (Gouveia et al., 2019), and Spanish (Bernabé-Valero et al., 2013; Langer et al., 2016). It was mostly possible to replicate the unidimensional structure of the GQ-6 and to provide similar reliabilities, e.g., the Hungarian version had a good reliability of Cronbach’s alphas between 0.75 and 0.79 (Tamás et al., 2014). However, some studies encountered difficulties to validate the factor structure of the GQ-6, since item 6 (“Long amounts of time can go by before I feel grateful to something or someone”) did not load satisfactory on the proposed factor. Thus, two studies found a better model fit for the Spanish version when removing item six (Bernabé-Valero et al., 2013; Langer et al., 2016). Similar results were found for the Chinese version (Chen et al., 2008). The Dutch version also reported poor fit of item 6, although it was retained, since a possible exclusion did not improve the overall model fit (Jans-Beken et al., 2015). A recent study found that a three item version (GQ-3) of the GQ-5 provides the best model fit for a sample of Filipino high school students (Valdez and Chu, 2018). Due to these mixed results concerning the factor structure of the GQ-6, we decided to include the 6 items form in our study and test whether the six or five item version fits better.

It was hypothesized that the one-factor model of the GQ-6 and the hierarchical structure of the MCGM could be replicated through confirmatory factor analyses for the German versions. In addition, construct validity was examined. We used a number of constructs that have been applied in previous studies such as subjective well-being and positive and negative affect (Morgan et al., 2017), empathy (Ding and Song, 2017), as well as life satisfaction and psychological symptoms (McCullough et al., 2002). In line with findings in existing literature, we expected positive correlations between gratitude and positive affect, well-being, empathy and negative relationships with negative affect, anxiety, and depression.

## Materials and Methods

### Participants and Procedure

A total of 508 participants (79% female, 20% male, 1% other) completed the questionnaire using an online survey tool. In return they received their gratitude score in comparison to the United Kingdom sample (Morgan et al., 2017). The link to the survey was distributed through various mailing lists of German universities and through Facebook groups that are associated with psychological studies. Participants’ age ranged from 18 to 67 years (*M* = 24.80, SD = 8.00). Their highest educational level was 7% primary education, 65% A-levels and 28% university degree, with about 30% being psychology students. The order of presentation of the scales was randomized and average time to complete was 17.30 min (SD = 6.79). Due to using an online survey tool with all questions had to be answered mandatorily, there occurred no missing data.

In addition, a sample with 1,599 participants (52% female) from across the United Kingdom was used to test cultural measurement invariance. It was carefully selected to reflect United Kingdom population estimates. Participants’ age ranged from 18 to 83 years (*M* = 51.43, SD = 12.96). This sample was used for the initial validation of the MCGM in the United Kingdom (Morgan et al., 2017).

### Measures

The German versions of the GQ-6 and the MCGM were derived using back-translation procedure. First, a translator was employed to translate the English versions into German. Thus, another professional translator retranslated these items into English. Eventually, both versions were consolidated by a psychologist and the final versions (GQ-6-G, MCGM-G) were later revised together with the authors to provide the best possible translation. The German items of both instruments are provided in Appendices A and B.

The GQ-6-G consists of six items that measure span, frequency, intensity and density of gratitude and primarily focuses on the unidimensional emotional component of gratitude. Participants have to rate six items (e.g., “I have so much in life to be thankful for”) on a seven-point Likert scale (1 = *strongly disagree* to 7 = *strongly agree*; GQ-6: α = 0.82; McCullough et al., 2002).

The MCGM-G consists of 43 items with four corresponding components of gratitude: (a) conceptions (or understandings) of gratitude; (b) grateful emotions; (c) attitudes toward gratitude; and (d) gratitude-related behaviors. For the conceptual component, participants are presented with seven scenarios involving different conceptions of gratitude: baseline, ulterior motive, cost to benefactor, non-realized benefit, malicious intent, value of benefit, and mixed emotions (e.g., *baseline*: “A colleague nominates you for an award at work. If you win, you will receive recognition of your hard work and a voucher”). After each scenario respondents have to indicate if they are grateful to this person on a five-point Likert scale (ARE ratings; 1 = *strongly disagree* to 5 = *strongly agree*; original α = 0.54) and state what degree of gratitude they would feel in each situation (DEGREE ratings; 0 = *not at all grateful* to 100 = *most grateful you could feel*; original α = 0.79). The *emotional* component consists of the subscale *Feelings of gratitude* and combines six items (e.g., “I feel grateful for the people in my life”) that are rated on a seven-point Likert scale (1 = s*trongly disagree* to 7 = s*trongly agree*; original α = 0.87). The *attitude* component is measured analogously and consists of the subscales *Attitudes to appropriateness* (six items, e.g., “I only show gratitude toward people who clearly intended to benefit me”; original α = 0.85) and *Attitude of gratitude* (six items, e.g., “I believe gratitude is an important value to have”; original α = 0.74). The *behavioral* component is measured on a seven-point Likert scale (1 = *never* to 7 = *more than once a day*) and consists of the subscales *Behavioral shortcomings* (four items, e.g., “I overlook how much I have to be grateful for”; original α = 0.82), *Rituals/noticing benefits* (five items, e.g., “I stop and think about all the things I am grateful for”; original α = 0.92), and *Expressions (of gratitude)* (four items, e.g., “I express thanks to those who help me”; original α = 0.79; Morgan et al., 2017).

In addition to these two gratitude measures, we used five instruments to measure construct validity:

Positive and negative affect was measured using the German version of the Positive and Negative Affect Schedule (PANAS; Watson et al., 1988; Krohne et al., 1996). The PANAS is based on the theory that positive and negative emotional states are not simply bipolar opposites but rather independent measures. It consists of 20 items that measure the general tendencies to experience positive (e.g., “excited”) and negative affect (e.g., “distressed”). Participants had to indicate on a five-point Likert scale (1 = *not at all* to 5 = *extremely*) how well each of the adjectives described “how they generally feel.” In previous studies (Krohne et al., 1996) the German scale has shown good values of internal consistency (α = 0.84 to α = 0.86).

Subjective well-being was assessed with the Subjective Happiness Scale (SHS; Lyubomirsky and Lepper, 1999; Bieda et al., 2017). The SHS consists of four items that measure global subjective happiness. Participants answered on a seven-point Likert scale whose wording of anchor points depended on the question (e.g., “Compared to most of my peers, I consider myself,” 1 = *less happy* to 7 = *more happy*). Internal consistency of the German scale was reported to be good with α = 0.87 (Bieda et al., 2017).

In addition, we measured life satisfaction using the German version of the Satisfaction with Life Scale (SWLS; Diener et al., 1985; Glaesmer et al., 2011). The SWLS consist of five items and measures the cognitive component of subjective well-being. Participants indicated agreement with items (e.g., “In most ways my life is close to ideal”) on a seven-point Likert scale (1 = *strongly disagree* to 7 = *strongly agree*). The internal consistency of the German scale lies within an excellent level of α = 0.92 (Glaesmer et al., 2011).

The German short version of the Brief Symptom Inventory (BSI; Derogatis and Spencer, 1982; Franke et al., 2011) was used to examine psychological symptoms among the participants. Respondents had to rate their general suffering from anxiety and depression symptoms on a five-point Likert scale (1 = *not at all* to 5 = *most intensive*). The internal consistency of the German scale has proven to be good with α = 0.84 (Franke et al., 2011).

To measure the disposition toward empathy, the Empathic Concern and Perspective-Taking subscales of the Interpersonal Reactivity Index (IRI; Davis and Oathout, 1987; Paulus, 2009) were used. Participants had to rate Empathic Concern items (e.g., “When I see someone being taken advantage of, I feel kind of protective toward them”) and Perspective-Taking items (e.g., “Before criticizing somebody, I try to imagine how I would feel if I were in their place”) on a five-point Likert scale (1 = *never* to 5 = *always*). Internal consistency of the German scales is acceptable (Empathic Concern: α = 0.77; Perspective-Taking: α = 0.77; Lauterbach and Hosser, 2007).

### Statistical Analysis

First, confirmatory factor analysis (CFA) was used in order to test the factor structure of the GQ-6-G and the MCGM-G for the German sample. As data were not normally distributed, robust maximum likelihood estimation (MLM) with Satorra and Bentler (2001) scaled χ^2^ was used. In order to obtain robust estimates of the fit indices, we also adjusted those for the robust χ^2^ test statistic (Walker and Smith, 2016). Evaluation of the model fit followed typical conventions: Ideally the χ^2^-test should not be significant (Schermelleh-Engel et al., 2003). However, the χ^2^-test tends to produce high and statistic significant values when sample size is high (*N* > 200: Walker and Smith, 2016; *N* > 250: Bühner, 2011). Thus, the ratio χ^2^ divided by degrees of freedom (χ^2^/*df*) represents a better fit index (Bentler and Bonett, 1980) and should be smaller than 3 (Kline, 1998). Values of the SRMR below 0.05 are good (Byrne, 1998) and should not exceed 0.08 (Hu and Bentler, 1999), though it must be noted that the SRMR decreases with larger sample size (Hooper et al., 2008). Values of the RMSEA below 0.05 indicate good model fit and also should not exceed 0.08 (Browne and Cudeck, 1993), whereas CFI and TLI should be above 0.95 (Schermelleh-Engel et al., 2003).

Second, multi-group comparisons (Vandenberg and Lance, 2000; Fischer and Karl, 2019) were conducted to assess measurement invariance between the German and United Kingdom sample. The analysis was based on the three typical phases described by Fischer and Karl (2019). In the first step, the baseline model is compared with the configural model to examine whether the overall factor structure holds up similarly for both groups (configural invariance). The next step is to test whether factor loadings are equivalent across the groups (metric invariance). The last step is to check whether the item intercepts are equivalent across groups (scalar invariance). In case full measurement invariance could not be established, partial invariance was examined (Byrne et al., 1989; Putnick and Bornstein, 2016). Thus, modification indices were used to check for non-invariant items. In order to obtain partial measurement invariance, at least half of the items of a factor should be equal across groups (Steenkamp and Baumgartner, 1998; Vandenberg and Lance, 2000). Evaluation of the model fit followed typical conventions: Since χ^2^ is sensitive to sample size, differences in the CFI (Little, 1997) and RMSEA (Little et al., 2007) are more informative and should be below 0.01 for each level of invariance (Cheung and Rensvold, 2002; Putnick and Bornstein, 2016; Fischer and Karl, 2019). For reporting results of the multi-group comparisons, we followed the suggestions of Putnick and Bornstein (2016).

Third, analysis of the factor structure and cultural invariance was followed by assessment of Cronbach’s Alpha (α) of the gratitude measures as well a check for normal distribution using the Shapiro-Wilk test. To ensure a good reliability, item-total-correlation should be above 0.30 (Bühner, 2011) and Cronbach’s α should be above 0.70 (Schermelleh-Engel and Werner, 2012).

Finally, the intercorrelations as well as criterion-related and discriminant validity were calculated.

Data were analyzed using R Studio (version 1.2.1335 on macOS, R version 4.0.0). The lavaan package (Rosseel, 2012) was used to calculate the CFAs. The cyc package (Karl, 2020) and semTools (Jorgensen, 2020) were used to conduct the cultural invariance analyses. The level of significance for all analyses was α = 5%.

## Results

### Factor Structure of GQ-6-G and MCGM-G

We first tested the one-factor structure of the GQ-6-G. Results revealed a mediocre model fit (see Table 1). Factor loading of the reverse-coded item 6 (“Long amounts of time can go by before I feel grateful to something or someone”) appeared to be low (0.29). Thus, we run a second analysis after excluding item 6 (GQ-5-G). All fit indices improved and were acceptable to good apart from the significant χ^2^ test (see Table 1).

**TABLE 1:** Goodness-of-fit indices of the CFA models (*N* = 508).

**Model**	**Fit indices**						
	
	**χ^2^ (*df*)^a^**	***p***	**χ^2^*/df***	**CFI**	**TLI**	**RMSEA [90% CI]**	**SRMR**
GQ-6-G	47.58(9)	<0.001	5.29	0.96	0.93	0.092 [0.069; 0.116]	0.052
GQ-5-G	18.16(5)	0.003	3.63	0.98	0.97	0.072 [0.041; 0.105]	0.027
MCGM-G	924.00(362)	<0.001	2.55	0.91	0.89	0.063 [0.058; 0.068]	0.075
MCGM-G^b^	712.49(309)	<0.001	2.31	0.93	0.92	0.058 [0.052; 0.064]	0.067
MCGM-G^c^	644.65(284)	<0.001	2.27	0.94	0.93	0.050 [0.046; 0.054]	0.059
MCGM-G SOF^d^	697.64(291)	<0.001	2.40	0.93	0.92	0.060 [0.055; 0.066]	0.069

*^*a*^χ^2^ is Satorra-Bentler corrected. ^*b*^Modified model without items 2 and 6 in the scale *Attitudes to appropriateness*. ^*c*^Modified model without items 1, 2, and 6 in the scale *Attitudes to appropriateness*. ^*d*^MCGM-G-SOF is based on modified version of MCGM-G without items 1, 2, and 6 in the scale *Attitudes to appropriateness*.*

Second, we tested the factor structure of the MCGM-G. Here, three competing models were calculated. Fit indices of the original six-factor model (Morgan et al., 2017) indicated good fit apart from the significant χ^2^ test. However, items 2 (“Gratitude should be reserved for when someone intends to benefit you”) and 6 (“I only feel grateful when the benefit is of genuine value to me”) of the *Attitude of appropriateness* scale turned out to have negative factor loadings. We checked whether there occurred any mistake with reverse-coding of these two items during the previously performed analysis. Since no error could be found, we decided to exclude the items. After eliminating both items, model fit increased regarding all fit indices (CFI = 0.93, TFI = 0.92, RMSEA = 0.058, SRMR = 0.067). Interestingly, item 1 of the same scale appeared to have some issues as well with a low corrected item-total correlation of *r*_*it*_ = 0.18. After the exclusion of item 1, the model fit further increased (CFI = 0.94, TFI = 0.93, RMSEA = 0.050, SRMR = 0.059). Lastly, the second-order factor model was analyzed. Here, results were slightly worse compared to the modified six-factor model but still in an acceptable to good range (see Table 1).

### Measurement Invariance Between Cultures of GQ-5-G and MCGM-G

The results of multi-group tests of measurement invariance of the GQ-5-G and the MCGM-G are presented in Tables 2 and 3.

**TABLE 2:** Summary of cultural invariance analyses between groups for the GQ-6-G and GQ-5-G.

**Model**	**Fit indices**									
	
	**χ^2^ (*df*)**	**CFI**	**RMSEA [90% CI]**	**SRMR**	**Model comp**	**Δχ^2^ (*df*)**	**ΔCFI**	**ΔRMSEA**	**ΔSRMR**	**Decision**
***GQ-6-G***										
Model A: Configural invariance	198.66(18)	0.960	0.098 [0.086–0.110]	0.039	–	–	–	–	–	–
Model B: Metric invariance	255.52(23)	0.948	0.098 [0.087–0.109]	0.058	A	56.86 (5)	0.012	0.000	0.019	Reject
Model C: Scalar invariance	328.27(28)	0.933	0.101 [0.091–0.111]	0.064	B	72.75 (5)	0.015	0.003	0.006	Reject
***GQ-5-G***										
Model A: Configural invariance	72.43(10)	0.984	0.077 [0.061–0.094]	0.020	–	–	–	–	–	–
Model B: Metric invariance	94.74(14)	0.980	0.074 [0.060–0.088]	0.031	A	22.31 (4)	0.004	0.003	0.011	Accept
Model C: Scalar invariance	132.67(18)	0.971	0.078 [0.066–0.090]	0.035	B	37.93 (4)	0.009	0.004	0.004	Accept

*All χ^2^ tests and Δχ^2^ were significant, *p* < 0.01.*

**TABLE 3:** Summary of cultural invariance analyses between groups for the MCGM-G and MCGM-G without items 1, 2, and 6 in the scale *Attitudes to appropriateness*.

**Model**	**Fit indices**									
	
	**χ^2^ (*df*)**	**CFI**	**RMSEA [90% CI]**	**SRMR**	**Model comp**	**Δχ^2^ (*df*)**	**ΔCFI**	**ΔRMSEA**	**ΔSRMR**	**Decision**
***MCGM-G***										
Model A: Configural invariance	2423.21(724)	0.946	0.047 [0.045–0.049]	0.044	–	–	–	–	–	–
Model B: Metric invariance	3143.37(747)	0.923	0.055 [0.053–0.057]	0.056	A	720.16 (23)	0.023	0.008	0.012	Reject
Model C: Scalar invariance	5426.85(770)	0.851	0.076 [0.074–0.078]	0.081	B	2.283.48 (23)	0.072	0.021	0.025	Reject
***MCGM-G*^*a*^**										
Model A: Configural invariance	1845.90(568)	0.955	0.046	0.039	–	–	–	–	–	–
Model B: Metric invariance	1998.34(588)	0.951	0.048 [0.045–0.050]	0.044	A	152.44 (20)	0.004	0.002	0.005	Accept
Model C: Scalar invariance	3025.90(608)	0.916	0.061 [0.059–0.064]	0.052	B	1.027.56 (20)	0.035	0.013	0.008	Reject
Model D: Partial scalar invariance^*b*^	2243.13(592)	0.942	0.052 [0.049–0.054]	0.046	B	244.79 (4)	0.009	0.009	0.002	Accept

*All χ^2^ tests and Δχ^2^ were significant, *p* < 0.01. ^*a*^Modified model without items 1, 2, and 6 in the scale *Attitudes to appropriateness*. ^*b*^Intercepts of the following items of each factor were allowed to vary between groups: FOG-i2, FOG-i4, FOG-i5, FOG-i6, ATA-i4, ATA-i5, BS-i2, BS-i3, RNB-i4, RNB-i5, EOG-i3, AOG-i1, AOG-i2, AOG-i3, AOG-i4. FOG, feelings of gratitude; ATA, attitudes to appropriateness; BS, behavioral shortcomings; RNB, rituals/noticing benefits; EOG, expression of gratitude; AOG, attitude of gratitude.*

Fit indices of the baseline model of the GQ-5-G indicate configural measurement invariance (χ^2^ = 72.43, *df* = 10, *p* < 0.001; CFI = 0.984; RMSEA = 0.077; SRMR = 0.020). A comparison with the model testing metric measurement invariance showed that the fit is worse (Δχ^2^ = 22.31, *df* = 4, *p* < 0.001). Since the χ^2^ difference test is sensitive to sample size and the other fit indices were good (CFI = 0.980; RMSEA = 0.074; SRMR = 0.031), metric measurement invariance can be assumed. Similar results were found for scalar measurement invariance. The χ^2^ difference indicated a worse fit (Δχ^2^ = 37.93, *df* = 4, *p* < 0.001). However, the other common fit indices were good (CFI = 0.971; RMSEA = 0.078; SRMR = 0.035), so scalar measurement invariance can be assumed.

Fit indices of the baseline model of the MCGM-G indicate configural measurement invariance (χ^2^ = 1845.90, *df* = 568, *p* < 0.001; CFI = 0.955; RMSEA = 0.046; SRMR = 0.039). A comparison with the model testing metric measurement invariance showed that the fit is worse (Δχ^2^ = 152.44, *df* = 20, *p* < 0.001). Since the χ^2^ difference test is sensitive to sample size and the other fit indices were good (CFI = 0.951; RMSEA = 0.048; SRMR = 0.044), metric measurement invariance can be assumed. However, results indicated a worse overall model fit on the level of scalar measurement invariance. In addition to the significant χ^2^ difference (Δχ^2^ = 1027.56, *df* = 20, *p* < 0.001), the differences of the other common fit indices were above the acceptable threshold of 0.01 (ΔCFI = 0.035; ΔRMSEA = 0.013). Thus, scalar measurement invariance cannot be assumed. Subsequent analyses using modification indices revealed that several item intercepts on all factors were not invariant across groups. Partial scalar measurement invariance could be established by allowing the intercepts of items 2, 4, 5, and 6 of Feelings of gratitude, items 4 and 5 of Attitudes to appropriateness, items 2 and 3 of Behavioral shortcomings, items 4 and 5 of Rituals/Noticing benefits, item 3 of Expression of gratitude and items 1, 2, 3, and 4 of Attitude of gratitude to vary between groups (χ^2^ = 2243.13, *df* = 592, *p* < 0.001; CFI = 0.942; RMSEA = 0.052; SRMR = 0.046; ΔCFI = 0.009; ΔRMSEA = 0.009; ΔSRMR = 0.002).

### Intercorrelations and Reliability

Shapiro-Wilk test indicated that the GQ-5-G and all dimensions (including second-order factors) of the MCGM-G are not normally distributed (*Skewness* = −1.09 to 0.20, *Kurtosis* = 2.80–4.05). The average corrected item–total correlation for the GQ-5-G was *r*_*it*_ = 0.49 and *r*_*it*_ = 0.33–0.62 for the MCGM-G, respectively. All first- and second-order factors of the MCGM-G and the GQ-5-G had acceptable to excellent internal consistencies (Cronbach’s α = 0.73–0.90; Table 4). A detailed overview can be found in Appendices C and D.

**TABLE 4:** Correlations between GQ-5-G and MCGM-G second-order factors and scales.

**Variable**	**M (SD)**	**α**	**1**	**2**	**3**	**4**	**5**	**6**	**7**	**8**
1. GQ-5-G	5.44 (1.03)	0.82	–	–	–	–	–	–	–	–
2. MCGM-G: Attitude	5.28 (0.70)	0.73	0.36** [0.28, 0.43]	–	–	–	–	–	–	–
3. MCGM-G: Behavior	4.54 (0.81)	0.84	0.45** [0.37, 0.51]	0.35** [0.27, 0.42]	–	–	–	–	–	–
4. Feelings of gratitude	5.52 (1.03)	0.90	0.81** [0.78, 0.84]	0.35** [0.27, 0.42]	0.46** [0.39, 0.53]	–	–	–	–	–
5. Attitudes to appropriateness	5.13 (1.22)	0.78	0.16** [0.07, 0.24]	0.86** [0.84, 0.88]	0.18** [0.10, 0.26]	0.16** [0.08, 0.25]	–	–	–	–
6. Behavioral shortcomings	3.86 (1.38)	0.87	0.18** [0.09, 0.26]	0.20** [0.11, 0.28]	0.71** [0.67, 0.75]	0.18** [0.10, 0.26]	0.18** [0.09, 0.26]	–	–	–
7. Rituals/noticing benefits	4.50 (1.01)	0.88	0.51** [0.45, 0.57]	0.26** [0.17, 0.34]	0.75** [0.71, 0.79]	0.51** [0.44, 0.57]	0.11* [0.03, 0.20]	0.25** [0.17, 0.33]	–	–
8. Expressions (of gratitude)	5.27 (1.02)	0.79	0.31** [0.23, 0.39]	0.30** [0.22, 0.38]	0.66** [0.61, 0.71]	0.34** [0.26, 0.41]	0.08 [−0.01, 0.16]	0.09*[0.00, 0.17]	0.45** [0.37, 0.51]	–
9. Attitude of gratitude	6.12 (0.84)	0.76	0.45** [0.38, 0.52]	0.69** [0.65, 0.74]	0.40** [0.33, 0.47]	0.45** [0.38, 0.52]	0.23** [0.14, 0.31]	0.11*[0.02, 0.19]	0.32** [0.24, 0.40]	0.48** [0.41, 0.55]

*M* and SD are used to represent mean and standard deviation, respectively. α refers to Cronbach’s α. Values in square brackets indicate the 95% confidence interval for each correlation. The confidence interval is a plausible range of population correlations that could have caused the sample correlation (Cumming, 2014). * Indicates *p* < 0.05. ** indicates *p* < 0.01.

The GQ-5-G significantly correlated with all first- and second-order factors of the MCGM-G. The strongest relationship refers to the GQ-5-G and the emotional factor of the MCGM-G (*r* = 0.81, *p* < 0.01).

### Criterion-Related and Discriminant Validity

The correlations between the gratitude scales (GQ-5-G, MCGM-G) and the criterion-related variables are presented in Tables 5, 6. As expected, we can report significant positive correlations between the gratitude measures and positive affect, well-being as well as empathy scales. The strongest relationship was found between the GQ-5-G and life satisfaction (*r* = 0.51, *p* < 0.01). In addition, significant negative correlations can be reported for depression and anxiety measures as well as negative affect.

**TABLE 5:** Correlations between GQ-5-G and criterion-related scales.

**Variable**	**1**	**2**	**3**	**4**	**5**	**6**	**7**	**8**
1. GQ-5-G	–	–	–	–	–	–	–	–
2. Life satisfaction	0.51** [0.44, 0.57]	–	–	–	–	–	–	–
3. Subjective well-being	0.41** [0.34, 0.48]	0.55** [0.49, 0.61]	–	–	–	–	–	–
4. Positive affect	0.35** [0.27, 0.42]	0.41** [0.34, 0.48]	0.50** [0.43, 0.56]	–	–	–	–	–
5. Negativeaffect	−0.27** [−0.35, −0.19]	−0.42** [−0.49, −0.35]	−0.34** [−0.41, −0.26]	−0.13** [−0.21, −0.04]	–	–	–	–
6. Empathic concern	0.28** [0.20, 0.36]	0.05 [−0.03, 0.14]	0.08 [−0.01, 0.17]	0.17** [0.08, 0.25]	0.05 [−0.04, 0.13]	–	–	–
7. Perspective taking	0.26** [0.18, 0.34]	0.20** [0.12, 0.29]	0.26** [0.18, 0.34]	0.16** [0.07, 0.24]	−0.18** [−0.26, −0.09]	0.39** [0.31, 0.46]	–	–
8. Depression	−0.38** [−0.45, −0.30]	−0.58** [−0.64, −0.52]	−0.52** [−0.58, −0.45]	−0.43** [−0.50, −0.35]	0.60** [0.54, 0.65]	0.03 [−0.05, 0.12]	−0.15** [−0.23, −0.06]	–
9. Anxiety	−0.22** [−0.30, −0.13]	−0.43** [−0.50, −0.35]	−0.34** [−0.41, −0.26]	−0.22** [−0.30, −0.14]	0.63** [0.57, 0.68]	0.06 [−0.03, 0.14]	−0.17** [−0.26, −0.09]	0.65** [0.60, 0.70]

Values in square brackets indicate the 95% confidence interval for each correlation. The confidence interval is a plausible range of population correlations that could have caused the sample correlation (Cumming, 2014). * Indicates *p* < 0.05. ** indicates *p* < 0.01.

**TABLE 6:** Correlations between MCGM-G second-order factors and criterion-related scales.

**Variable**	**1**	**2**	**3**	**4**	**5**	**6**	**7**	**8**	**9**	**10**
1. MCGM-G: attitude	–	–	–	–	–	–	–	–	–	–
2. MCGM-G: behavior	0.35** [0.27, 0.42]	–	–	–	–	–	–	–	–	–
3. MCGM-G: emotion	0.35** [0.27, 0.42]	0.46** [0.39, 0.53]	–	–	–	–	–	–	–	–
4. Life satisfaction	0.21** [0.12, 0.29]	0.31** [0.23, 0.39]	0.49** [0.43, 0.56]	–	–	–	–	–	–	–
5. Subjective well-being	0.18** [0.10, 0.26]	0.35** [0.27, 0.42]	0.45** [0.37, 0.51]	0.55** [0.49, 0.61]	–	–	–	–	–	–
6. Positive affect	0.11* [0.02, 0.19]	0.39** [0.32, 0.47]	0.33** [0.25, 0.41]	0.41** [0.34, 0.48]	0.50** [0.43, 0.56]	–	–	–	–	–
7. Negative affect	−0.25** [−0.33, −0.16]	−0.21** [−0.29, −0.12]	−0.24** [−0.32, −0.16]	−0.42** [−0.49, −0.35]	−0.34** [−0.41, −0.26]	−0.13** [−0.21, −0.04]	–	–	–	–
8. Empathic concern	0.14** [0.05, 0.22]	0.35** [0.27, 0.43]	0.31** [0.22, 0.38]	0.05 [−0.03, 0.14]	0.08 [−0.01, 0.17]	0.17** [0.08, 0.25]	0.05 [−0.04, 0.13]	–	–	–
9. Perspective taking	0.20** [0.11, 0.28]	0.32** [0.24, 0.39]	0.27** [0.18, 0.35]	0.20** [0.12, 0.29]	0.26** [0.18, 0.34]	0.16** [0.07, 0.24]	−0.18** [−0.26, −0.09]	0.39** [0.31, 0.46]	–	–
10. Depression	−0.20** [−0.28, −0.12]	−0.27** [−0.35, −0.19]	−0.37** [−0.44, −0.29]	−0.58** [−0.64, −0.52]	−0.52** [−0.58, −0.45]	−0.43** [−0.50, −0.35]	0.60** [0.54, 0.65]	0.03 [−0.05, 0.12]	−0.15** [−0.23, −0.06]	–
11. Anxiety	−0.18** [−0.26, −0.09]	−0.17** [−0.26, −0.09]	−0.21** [−0.30, −0.13]	−0.43** [−0.50, −0.35]	−0.34** [−0.41, −0.26]	−0.22** [−0.30, −0.14]	0.63** [0.57, 0.68]	0.06 [−0.03, 0.14]	−0.17** [−0.26, −0.09]	0.65** [0.60, 0.70]

Values in square brackets indicate the 95% confidence interval for each correlation. The confidence interval is a plausible range of population correlations that could have caused the sample correlation (Cumming, 2014). * Indicates *p* < 0.05. ** indicates *p* < 0.01.

## Discussion

The main aim of the current study was to validate the psychometric properties of two gratitude measures in German language (GQ-6-G and the MCGM-G). We first sought to establish well-fitting baseline models for the two instruments conducting CFAs. The next step was to check for cultural measurement invariance using multi-group comparisons.

We replicated the one-factor structure of the GQ-6 and found a good fit after excluding item 6 of the scale. Hence, the German version of the GQ-6 enquires the same problem with item 6 as already reported for the Dutch (Jans-Beken et al., 2015) or Spanish version (Langer et al., 2016). The authors of the Spanish version argue that the five-item version is especially appropriate for younger populations (university students and adolescents) in comparison to older participants (Langer et al., 2016). Similar results were found in a non-Western sample examining gratitude among Filipino high school students (Valdez et al., 2017). Considering the mean age of just about 25 years (SD = 8.00) in the present study, this could also be a reasonable explanation for our findings. Multi-group comparisons were conducted to test measurement invariance across two samples from United Kingdom and Germany. Thus, measurement invariance on all three levels (configural, metric, and scalar) can be assumed for the GQ-5-G but not for the GQ-6-G, since differences in the CFI and RMSEA for the latter were above the threshold of 0.01. These results indicate that participants from both countries conceptualize the one-factor structure of the GQ-5-G in the same way (configural invariance). As metric invariance was also supported, associations between the GQ-5-G and other variables can be compared across samples from United Kingdom and Germany. Eventually, scalar invariance suggests that mean differences between groups are due to differences in the latent construct which allows comparisons of mean differences. We therefore recommend using the five-item version (GQ-5-G) for future research.

We found a good model fit for the MCGM-G after excluding three items of the Attitudes to appropriateness scale (items 1, 2, and 6 were excluded). Interestingly, the deviations compared to the original version of the MCGM only referred to this dimension. As Attitudes to appropriateness captures the degree when gratitude is and is not warranted, the findings might suggest different cultural understandings. Further studies should therefore explore this dimension of gratitude in a more in-depth examination. However, results of the CFAs did not reveal superior model fit of the hierarchical structure of the MCGM-G with second-order factors, as fit indices were almost identical compared to the six-factor solution. In contrast to the original study, both models had slightly worse fit. Thus, future studies should analyze the underlying factor structure more closely comprising larger samples. Nevertheless, on the basis of current knowledge, it seems appropriate to use the second-order factors of MCGM-G, if an economic consideration or merely a comparison of attitude and behavior components of gratitude is required. Multi-group comparisons of the MCGM-G testing for cultural invariance revealed that configural invariance was established. Thus, similar latent factors were present in both countries suggesting that participants from both samples conceptualize the six components of the MCGM-G similarly. Metric invariance was also supported. Accordingly, the factor structure of the six dimensions was equivalent across both groups indicating that individuals attributed the same meaning to the latent constructs. Since metric invariance was established, associations between the MCGM-G and other variables can be compared across samples from United Kingdom and Germany. However, scalar measurement invariance could not be supported. In total, 15-item intercepts on all factors turned out not to be equal across the two samples. When these item intercepts were allowed to vary between the two groups, partial measurement invariance could be obtained. An exception is the dimension Attitude of gratitude since all item intercepts of this factor were non-invariant. Thus, latent mean comparisons for five MCGM-G factors can be conducted, if the corresponding items are allowed to have their own intercept.

The strong correlation between the emotion component of the MCGM-G and the GQ-5-G (*r* = 0.81, *p* < 0.001) is even higher than in previous studies (e.g., *r* = 0.71, *p* < 0.001; Morgan et al., 2017). This supports the assumption that the GQ-5-G only taps feelings of gratitude, as it was already suggested by Morgan et al. (2017). Thus, all correlations between the other factors of the MCGM-G and the GQ-5-G were lower (*r* = 0.11 to *r* = 0.51).

Construct validity with criterion-related scales of both the GQ-5-G and the MCGM-G showed the expected correlations as already reported in previous studies (e.g., McCullough et al., 2002; Breen et al., 2010; Morgan et al., 2017). Overall, gratitude is associated with increased positive and lower negative outcomes. Wellbeing and affect scales revealed medium to strong effects, while effects of empathy, anxiety and depression scales can be characterized as low to medium effects. The associations between the criterion-related scales and the GQ-5-G and the MCGM-G show a similar pattern. However, the MCGM-G yields a more diverse perspective. Thus, our results indicate that the emotional component of gratitude is more strongly related to life satisfaction and subjective well-being, whereas the behavioral component is most strongly associated with positive affect and empathy. The correlations between the attitude component and the criterion-related scales are rather low.

## Limitations

As in many psychological studies, the German sample is biased toward female participants and has a low mean age of 24.80 years. This means that the conceptualization of gratitude may only be representative of young females. Although this seems to be a typical bias that also affects other validation studies of gratitude measures (e.g., Gouveia et al., 2019) as well as the original study of the GQ-6 by McCullough et al. (2002), we checked for measurement invariance between gender. The results revealed full measurement invariance across the gender of the participants for the GQ-5-G and the MCGM-G (see Appendix E). Thus, mean comparisons between genders on the latent factors can be analyzed. Nevertheless, future studies should comprise more representative samples regarding age and gender and analyze whether the factor structure reported here is stable across gender-balanced groups. It should also be mentioned that using maximum-likelihood estimations on data that violate multivariate normality can bias results. However, we tried to account for this limitation by using robust maximum likelihood estimation (MLM) with Satorra and Bentler (2001) scaled χ^2^ as well as robust estimates of the fit indices following the adjustments of Walker and Smith (2016). Overall, our results are based on only two countries. Therefore, future studies are needed to evaluate measurement invariance of the GQ5-G and the MCGM-G in other countries.

## Conclusion

The GQ-5-G and the MCGM-G are both reliable and valid instruments for measuring gratitude in Germany. Measurement invariance was established for the GQ-5-G and partial measurement invariance for five of six factors of the MCGM-G, respectively. Psychological research can rely on these tools for future studies on gratitude. In this context, the GQ-5-G can be considered a very good and economic choice if a reliable and valid instrument is needed to measure the emotional component of gratitude. In contrast, the multi-component approach of the MCGM-G offers a more diverse perspective on gratitude.

## Data Availability Statement

The datasets presented in this study can be found in an online repository in the Open Science Framework at https://osf.io/8v5ej/.

## Ethics Statement

Ethical review and approval was not required for the study on human participants in accordance with the local legislation and institutional requirements. The patients/participants provided their written informed consent to participate in this study.

## Author Contributions

NB and EL designed the studies and supervised the execution. NB collected the data. MH did the analysis. NB, EL, BM, and MH wrote the manuscript. All authors read and approved the final manuscript.

## Conflict of Interest

The authors declare that the research was conducted in the absence of any commercial or financial relationships that could be construed as a potential conflict of interest.
