# Supplementary material for: Measuring Gratitude in Germany: Validation Study of the German Version of the Gratitude Questionnaire-Six Item Form (GQ-6-G) and the Multi-Component Gratitude Measure (MCGM-G)
Source: Front Psychol. 2020 Oct 8;11:590108. doi: 10.3389/fpsyg.2020.590108 (PMC7586006; doi:10.3389/fpsyg.2020.590108)
Supplement: Supplementary file 1 [file Data_Sheet_1.pdf]

# 1 Appendix

**Appendix A.** Items of the English and German version of the GQ-6.

| Item no. | GQ-6 (English, McCullough et al., 2002) | GQ-6-G (German) |
|---|---|---|
| 1 | I have so much in life to be thankful for. | Es gibt so viel in meinem Leben, für das ich dankbar bin. |
| 2 | If I had to list everything that I felt grateful for, it would be a very long list. | Wenn ich alles auflisten müsste, für das ich dankbar bin, wäre dies eine sehr lange Liste. |
| 3 | When I look at the world, I don't see much to be grateful for.(*) | Wenn ich die Welt betrachte, dann sehe ich nicht viel, für das ich dankbar bin.(*) |
| 4 | I am grateful to a wide variety of people. | Ich bin einer Vielzahl an Menschen dankbar. |
| 5 | As I get older I find myself more able to appreciate the people, events, and situations that have been part of my life history. | Je älter ich werde, desto mehr bin ich in der Lage, für die Menschen, die Ereignisse und die Situationen, die ein Teil meiner Lebensgeschichte gewesen sind, dankbar zu sein. |
| 6 | Long amounts of time can go by before I feel grateful to something or someone.(*) | Eine lange Zeit kann verstreichen, bis ich für etwas oder jemandem dankbar bin.(*) [a] |

[a] Item was excluded in GQ-5-G.

(*) Reverse scored items.

**Appendix B**. Items of the English and German version of the MCGM.

| Scale / item no. | MCGM (English, Morgan et al., 2017) | MCGM-G (German) |
|---|---|---|
| FOG / 1 | There are so many people that I feel grateful towards. | Es gibt so viele Menschen, denen ich dankbar bin. |
| FOG / 2 | There are so many people that I feel grateful for. | Es gibt so viele Menschen in meinem Leben, für die ich dankbar bin. |
| FOG / 3 | I feel appreciative of the support of many people in my life's journey. | Ich bin dankbar für die Unterstützung vieler Menschen, die mich auf meinem Lebensweg begleiten. |
| FOG / 4 | I feel grateful for the people in my life. | Ich bin dankbar für die Menschen in meinem Leben. |
| FOG / 5 | Thinking about all I have to be grateful for makes me feel happy. | Über alles nachzudenken, wofür ich dankbar bin, macht mich glücklich. |
| FOG / 6 | There are many things that I am grateful for. | Es gibt viele Dinge, für die ich dankbar bin. |
| ATA / 1 | Gratitude should be reserved for when someone does not want anything in return.(*) | Dankbarkeit sollte demjenigen entgegengebracht werden, der für seine Hilfe keine Gegenleistung erwartet. (*) [a] |
| ATA / 2 | Gratitude should be reserved for when someone intends to benefit you.(*) | Dankbarkeit sollte demjenigen entgegengebracht werden, der die Absicht hat, dir zu helfen. (*) [a] |
| ATA / 3 | I only show gratitude to people who have benefitted me without wanting anything in return.(*) | Ich bin nur den Menschen dankbar, die mir geholfen haben, ohne dafür eine Gegenleistung zu erwarten. (*) |
| ATA / 4 | I only show gratitude for the things that are not already due to me/are mine by right.(*) | Ich bin nur für die Dinge dankbar, die mir nicht ohnehin schon zustehen/mir gehören. (*) |
| ATA / 5 | I only show gratitude towards people who clearly intended to benefit me.(*) | Ich bin nur den Menschen dankbar, die ganz eindeutig nur das Beste für mich wollten.(*) |
| ATA / 6 | I only feel grateful when the benefit is of genuine value to me. | Ich bin nur dankbar, wenn der Nutzen einen echten Wert für mich hat.[a] |
| BS / 1 | I forget to let others know how much I appreciate them.(*) | Ich vergesse es, andere wissen zu lassen, wie sehr ich sie schätze. (*) |
| BS / 2 | I forget to reflect on the things that I am grateful for.(*) | Ich vergesse es, über die Dinge nachzudenken, für die ich dankbar bin. (*) |
| BS / 3 | I overlook how much I have to be grateful for.(*) | Ich bemerke nicht, wie viel ich habe, für das ich dankbar sein sollte. (*) |
| BS / 4 | I forget to remind myself that there is so much in life to be thankful for.(*) | Ich vergesse, mich selbst daran zu erinnern, dass es so viel im Leben gibt, für das ich dankbar sein sollte. (*) |
| RNB / 1 | I stop to recognize all the good things I have in my life. | Ich halte inne, um all die guten Dinge, die ich in meinem Leben habe, zu erkennen. |
| RNB / 2 | I recognize how many things I have to be grateful for. | Ich erkenne, wie viele Dinge es gibt, für die ich dankbar sein sollte. |

| | | |
|---|---|---|
| RNB / 3 | I stop and think about all the things I am grateful for. | Ich halte inne und denke über all die Dinge nach, für die ich dankbar bin. |
| RNB / 4 | I reflect on all the good things I have. | Ich denke über all die guten Dinge nach, die ich habe. |
| RNB / 5 | I remind myself of the benefits I have received. | Ich erinnere mich an die Unterstützung, die ich erhalten habe. |
| EOG / 1 | I make it a priority to thank others. | Ich mache es zu meiner Priorität, mich bei anderen zu bedanken. |
| EOG / 2 | I express thanks to those who help me. | Ich bedanke mich bei denjenigen, die mir helfen. |
| EOG / 3 | I notice the people who are kind to me. | Ich nehme die Menschen zur Kenntnis, die freundlich zu mir sind. |
| EOG / 4 | I go out of my way to thank others for their help. | Ich scheue keine Mühe, um anderen für ihre Hilfe zu danken. |
| AOG / 1 | I don't think it is necessary to show your gratitude to others.(*) | Ich denke nicht, dass es nötig ist, anderen seine Dankbarkeit zu zeigen. (*) |
| AOG / 2 | I believe it is important to thank people sincerely for the help they give me. | Ich glaube, dass es wichtig ist, Menschen für ihre Hilfe aufrichtig zu danken. |
| AOG / 3 | I believe gratitude is an important value to have. | Ich glaube, Dankbarkeit ist ein wichtiger Wert, den man haben sollte. |
| AOG / 4 | It is important to acknowledge the kindness of other people. | Es ist wichtig, die Freundlichkeit anderer Menschen zu schätzen zu wissen. |

[a] Items were excluded in final version MCGM-G.

[*] Reverse scored items.

FOG = Feelings of gratitude; ATA = Attitudes to appropriateness; BS = Behavioral shortcomings; RNB = Rituals/Noticing benefits; EOG = Expression of gratitude; AOG = Attitude of gratitude.

**Appendix C**. Item statistics of the GQ-5-G.

| Item no. | *M* (*SD*) | Item-total-correlation[a] | Cronbach's Alpha if item is deleted |
|---|---|---|---|
| 1 | 5.60 (1.18) | .75 | .75 |
| 2 | 5.33 (1.44) | .71 | .76 |
| 3 | 5.44 (1.49) | .43 | .84 |
| 4 | 5.31 (1.22) | .66 | .77 |
| 5 | 5.52 (1.35) | .57 | .80 |

[a] Part-whole corrected item-total-correlation.

**Appendix D**. Item statistics of the MCGM-G.

| Scale / Item no. | *M* (*SD*) | Item-total-correlation[a] | Cronbach's Alpha if item is deleted |
|---|---|---|---|
| FOG / 1 | 5.37 (1.25) | .79 | .88 |
| FOG / 2 | 5.62 (1.20) | .74 | .89 |
| FOG / 3 | 5.68 (1.22) | .78 | .88 |
| FOG / 4 | 5.77 (1.15) | .74 | .88 |
| FOG / 5 | 5.10 (1.48) | .64 | .90 |
| FOG / 6 | 5.55 (1.23) | .75 | .88 |
| | | | |
| ATA/ 3 | 5.05 (1.43) | .63 | .69 |
| ATA/ 4 | 5.55 (1.42) | .60 | .72 |
| ATA/ 5 | 4.79 (1.53) | .62 | .70 |
| | | | |
| BS / 1 | 4.01 (1.45) | .58 | .88 |
| BS / 2 | 3.60 (1.64) | .78 | .81 |
| BS / 3 | 3.96 (1.72) | .78 | .81 |
| BS / 4 | 3.86 (1.70) | .76 | .82 |
| | | | |
| RNB / 1 | 4.30 (1.26) | .72 | .86 |
| RNB / 2 | 4.70 (1.22) | .75 | .85 |
| RNB / 3 | 4.25 (1.27) | .81 | .84 |
| RNB / 4 | 4.51 (1.22) | .80 | .84 |
| RNB / 5 | 4.76 (1.14) | .54 | .90 |
| | | | |
| EOG / 1 | 4.46 (1.44) | .57 | .77 |
| EOG / 2 | 5.55 (1.21) | .69 | .71 |
| EOG / 3 | 5.96 (1.10) | .55 | .77 |
| EOG / 4 | 5.10 (1.40) | .65 | .72 |
| | | | |
| AOG / 1 | 6.14 (1.31) | .37 | .84 |
| AOG / 2 | 6.01 (1.09) | .63 | .68 |
| AOG / 3 | 6.12 (1.02) | .68 | .65 |
| AOG / 4 | 6.21 (0.95) | .66 | .67 |

[a] Part-whole corrected item-total-correlation.

**Appendix E**. Summary of measurement invariance analyses between gender for the GQ-5-G and MCGM-G.

| Model | Fit indices | | | | | | | |
|---|---|---|---|---|---|---|---|---|
| | $\chi^2(df)$ | *CFI* | *RMSEA* [90% CI] | *SRMR* | Model comp | $\Delta\chi^2(df)$ | *ΔCFI* | *ΔRMS*[illegible] |
| *GQ-5-G* | | | | | | | | |
| Model A: Configural invariance | 28.81 (10) | 0.981 | 0.086 [0.050-0.124] | 0.026 | -- | -- | -- | -- |
| Model B: Metric invariance | 30.40 (14) | 0.983 | 0.068 [0.034-0.101] | 0.032 | A | 1.59 (4) | 0.002 | 0.018 |
| Model C: Scalar invariance | 31.89 (18) | 0.986 | 0.055 [0.021-0.086] | 0.033 | B | 1.49 (4) | 0.003 | 0.013 |
| *MCGM-G*[a] | | | | | | | | |
| Model A: Configural invariance | 1258.92 (568) | 0.902 | 0.069 [0.064-0.074] | 0.062 | -- | -- | -- | -- |
| Model B: Metric invariance | 1277.99 (588) | 0.902 | 0.068 [0.063-0.073] | 0.064 | A | 19.07 (20) | 0.000 | 0.001 |
| Model C: Scalar invariance | 1330.36 (608) | 0.897 | 0.069 [0.064-0.074] | 0.064 | B | 52.37 (20) | 0.005 | 0.001 |

*Note.* All $\chi^2$ tests and $\Delta\chi^2$ were significant, $p < .01$.

[a] Modified model without items 1, 2, and 6 in the scale *Attitudes to appropriateness*
